# Self-Healing Capacity of Asphalt Mixtures Including By-Products Both as Aggregates and Heating Inductors

**DOI:** 10.3390/ma11050800

**Published:** 2018-05-15

**Authors:** Marta Vila-Cortavitarte, Daniel Jato-Espino, Daniel Castro-Fresno, Miguel Á. Calzada-Pérez

**Affiliations:** 1 GITECO Research Group, Universidad de Cantabria, 39005 Santander, Spain; jatod@unican.es (D.J.-E.); castrod@unican.es (D.C.-F.); 2 GCS Research Group, Universidad de Cantabria, 39005 Santander, Spain; calzadam@unican.es

**Keywords:** self-healing, asphalt mixtures, induction heating, magnetic induction, metallic by-products

## Abstract

Major advances have been achieved in the field of self-healing by magnetic induction in which the addition of metallic particles into asphalt mixtures enables repairing their own cracks. This technology has already been proven to increase the life expectancy of roads. Nevertheless, its higher costs in comparison with conventional maintenance caused by the price of virgin metallic particles still makes it unattractive for investment. This research aimed at making this process economically accessible as well as environmentally efficient. To this end, an intense search for suitable industrial by-products to substitute both the virgin metal particles and the natural aggregates forming asphalt mixtures was conducted. The set of by-products used included sand blasting wastes, stainless shot wastes, and polished wastes as metallic particles and other inert by-products as aggregates. The results demonstrated that the by-products were adequately heated, which leads to satisfactory healing ratios in comparison with the reference mixture.

## 1. Introduction

Self-healing is the process whereby asphalt can partially recover from damage and, therefore, produce an increase in the lifetime of roads and can limit the need for their maintenance. In other words, self-healing provides an opportunity to reduce road disruptions and attenuate the economic and environmental impacts stemming from the natural resources required to undertake traditional maintenance practices [1]. However, this process only happens innately in the absence of traffic loads and under ideal conditions in terms of temperature, which enables bitumen flowing to fill small cracks caused by aging phenomena [2]. The incorporation of different additives into asphalt mixtures contributes toward overcoming these limitations and improving their healing capacity. These catalyzers include encapsulated rejuvenators, nanoparticles, and metallic particles heated via either electromagnetic induction or microwaves [3]. Among them, induction heating has been found to be the most widely used approach for healing asphalt mixtures [4]. This technique consists of the resistance of conductive materials against cross Eddy currents induced by a magnetic field. In the context of asphalt mixtures, the process of magnetic induction only leads to the heating of the metallic particles performing as additives and having no effect on bitumen or aggregates [5]. 

The healing capacity of asphalt mixtures has become a widely researched topic over the last decade especially during the last three years. Liu et al. [6] examined the capacity of porous asphalt mixtures with steel fibers and wool to be heated via induction. Long steel wool with a small diameter was found to be more effective than short steel fibers with a large diameter to enhance the electrical conductivity of the mixtures. In contrast, the results obtained by García et al. [7] from 25 dense asphalt concrete mixtures with different lengths, quantities, and amounts of steel wool fibers suggested that the induction heating of the mixtures was enhanced using short fibers with large diameters. One year later, García et al. [8] extended the addition of steel wool fibers to porous asphalt. The healing rates of the mixtures were limited to 78% due to their deformation as a result of the heating process. Menozzi et al. [9] studied the reparation of microcracks caused by fatigue damage in dense asphalt mixtures including cast steel particles. The results demonstrated that the healing of the samples via induction heating enabled the extension of the lifetime of the samples up to 31%. Bueno et al. [10] subjected a series of up-scaled asphalt slabs with cast iron particles to different loading cycles, which proves that the application of induction heating at the initiation of the cracks produced and enabled the recovery of the original performance of the specimens. Norambuena-Contreras and Garcia [11] compared the healing degree of dense asphalt mixtures with steel wool fibers by microwave and induction heating, which means that the former resulted in a better crack closure due to the higher temperature of the binder. Zhu et al. [12] applied micro-wave heating to a Stone Mastic Asphalt (SMA-13) mixture containing Ni-Zn ferrite powder, which was proven to significantly improve the healing ability of the specimens tested. Franesqui et al. [13] found that the cracks of different asphalt mixtures, such as Asphalt Concrete (AC), Béton Bitumineux Minces (BBTM) and Porous Asphalt (PA), were completely closed when heating several industrial wastes (steel wool, steel filing, and metallic powder) via microwaves. 

In light of the target of these studies and the costs and emissions involved by using virgin metallic particles, this research aimed at assessing the healing capacity of asphalt mixtures containing different industrial by-products. This proves that these materials could be used as a substitute for raw materials when heated under magnetic induction. Therefore, the underlying goal sought was the valorization of a series of potentially conductive by-products including metallic particles in order to improve the viability of the healing process of asphalt mixtures in comparison with conventional maintenance practices by increasing its investment attractiveness and environmental efficiency.

## 2. Materials and Methods

**Figure 1:**
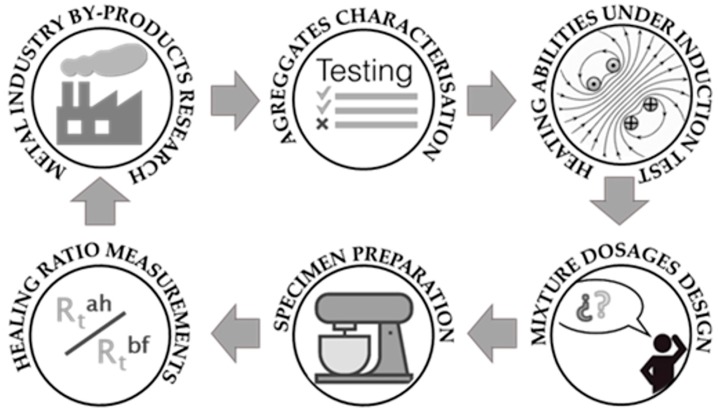
Flowchart of the six stages forming the proposed methodology.

The accomplishment of the objectives established in this research was approached by following the six steps shown in Figure 1. The first step consisted of contacting local metal industries to ask about their processes, their by-products, and potential collaboration by giving some samples for testing. Afterward, the collected by-products were fully characterized in laboratory including a heating under induction test over the metallic-based by-products to check the temperature they could achieve. The selection of the most suitable materials and the dosage of mixtures formed the fourth step, which was followed by the specimen preparation. Lastly, the calculation of a healing ratio by comparing the fracture resistance before and after healing illustrated the suitability of each experimental mixture. Steps three to six could be repeated at different times to optimize mixture designs in order to reach better healing ratios.

The mixture designed for the study was a dense asphalt concrete (AC-16) made of ophite as a coarse aggregate, limestone as a fine aggregate, and ferromagnetic particles to make the induction process possible. Both aggregates and ferromagnetic particles could be changed by one or more of the tested by-products. 

**Figure 2:**
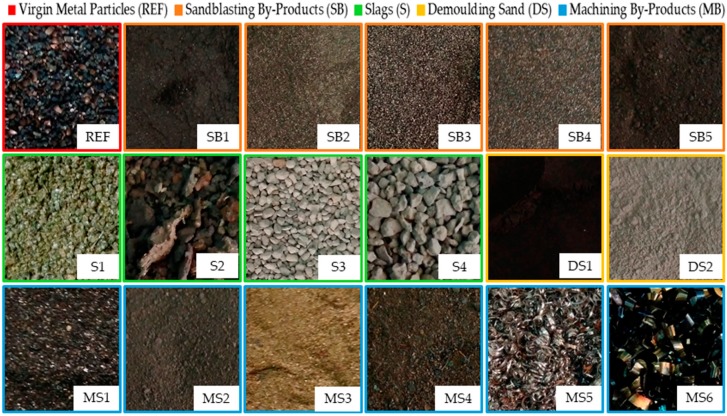
Textures of the set of by-products collected from local metal industries.

The industrial by-products used for the replacement of raw materials are included in Figure 2. Nine different local industries provided samples of their by-products. As a result, 17 different by-products were provided to the laboratory, which were divided into four groups for simplification purposes. This includes by-products coming from sandblasting processes (SBx), slags from different processes (Sx), demolding sands (DSx), and by-products from machining processes (MBx). In addition, virgin metal particles for shooting (Virgin Metal Particles, REF) were used as a reference for the metallic by-products.

In particular, the by-products shown in Figure 2 were blasting shoot wastes (SB1 and SB4), steel grit wastes (SB2 and SB3), sand wastes from sandblasting processes (SB5), green foundry slags (S1), bronze foundry slags (S2), arc-electric foundry slags (S3 and S4), demolding sand from different foundries (DS1 and DS2), unburnt foundry wastes (MB1), foundry ashes (MB2), bronze powder from polishing (MB3), deburring wastes (MB4), and machining shavings (MB5 and MB6). It is worth mentioning that the by-products were used in a straightforward manner from the industry as long as possible without treating them in any way to prevent overshadowing their potential economic and/or environmental benefits derived from their valorization.

### 2.1. Laboratory Tests

Once in the laboratory, the materials were characterized according to the Particles Size Gradation (EN 933-1) standard [14] in order to determine the particle size distributions required for dosage mixtures and in order to obtain a fraction that could be substituted by the by-products. The specific weight (g/cm^3^) of the materials was also calculated using the pycnometer method. Given the metallic nature of many of the by-products used, this test was developed with methanol instead of water in order to avoid their oxidation and the potential distortion of results. 

**Figure 3:**
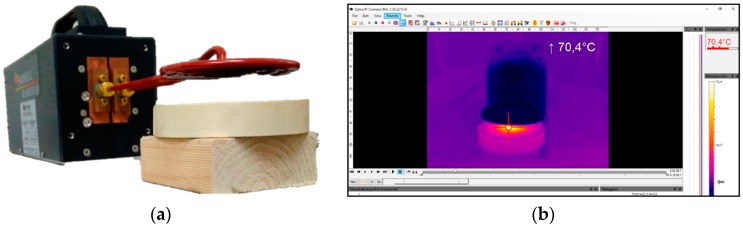
(**a**) By-products heating test; (**b**) Thermographic image recorded with the infrared camera.

In the case of the metallic by-products, an ad-hoc experiment was designed to check their suitability to be used as heating inductors in the healing process. To this end, a round wooden box was filled with the by-product and placed at 2 cm from the coil (see Figure 3a). An induction of 100 A intensity was applied over 3 min using the EASYHEAT 3542 system [15]. Both the heating time and the cooling time (15 extra minutes) were registered by an Optris PI Connect infrared thermometer-based camera [16] (see Figure 3b).

Based on the results of the particle size distribution and the heating performance tests, the most suitable materials were selected to compound the experimental mixtures. The temperature they reached in comparison with that achieved by the REF particles helped support this choice.

### 2.2. Mixture Dosage Design and Specimen Preparation

Taking into account the huge differences among the by-products used, the dosage methods for the replacement of raw materials by the by-products made by weight or volume were discarded [17,18]. Instead, dosages were undertaken seeking a continue particle size distribution of an asphalt concrete (AC-16) (see Table 1). This premise implies that the amount of material the mixture could accept without relevant deviations of the spindle was determined by the particle size distribution itself. Therefore, each mixture was comparable with the others although the amount of materials substituted was always different. The minimum bitumen content was calculated based on the Spanish Standard EN 13108-1 [19], which proposes a minimum of 4.5% in mixtures with an aggregate density of 2.65 g/cm^3^. According to the density ρ of the aggregates of the experimental mixtures, the reduced bitumen quantity (rbq) was determined by applying Equation (1).

(1) $$rbq\left(\%\right)=4.5\cdot \frac{2.65\;g/cm^{3}}{\rho \;g/cm^{3}}$$

Regarding the specimen preparation, the materials were combined in a laboratory mixer. Aggregates and bitumen were heated to 170 °C and 155 °C, respectively. The materials were added to the drum in the following order: coarse aggregate, fine aggregate plus metal particles, and bitumen. After a minute of mixing, the filler was added and all the materials were mixed together over 4 extra minutes. Metallic particles were mixed after heating with the fine aggregates in order to ensure a better distribution in the mixtures.

**Figure 4:**
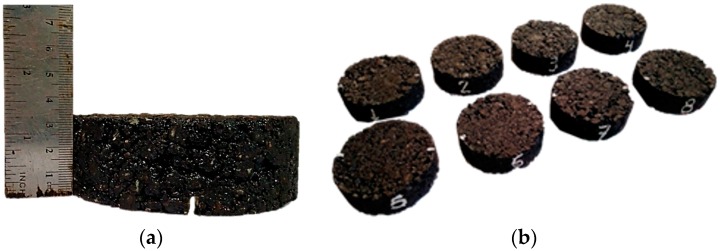
(**a**) Pre-notched sample; (**b**) Group of demolded specimens.

The samples prepared were half-height Marshall specimens (101.6 mm diameter and 33 mm height) compacted with 40 blows on each side. This reduction in the size of the samples was carried out with the objective of saving materials. In order to facilitate the compaction of these half-height specimens, a wooden wedge was introduced at the bottom of the mould to compensate for the reduced amount of material and keep the height from which the impact was produced. The specimens were pre-notched to a depth and width of 0.5 cm in the laboratory in order to facilitate obtaining a straight crack when the specimens were broken without compromising the healing surface, which was large enough to enable their subsequent testing. Figure 4 shows a group of demoulded specimens with the dimensions mentioned above and the details of a pre-notched specimen. 

### 2.3. Healing Measurements Using a Break-Heal-Break Test

This test consisted of breaking the pre-notched specimens both before and after subjecting them to a magnetic induction field. The result of the test was the healing ratio (HR), which is a percentage of remaining resistance in the specimens after being completely broken and healed by magnetic induction. 

**Figure 5:**
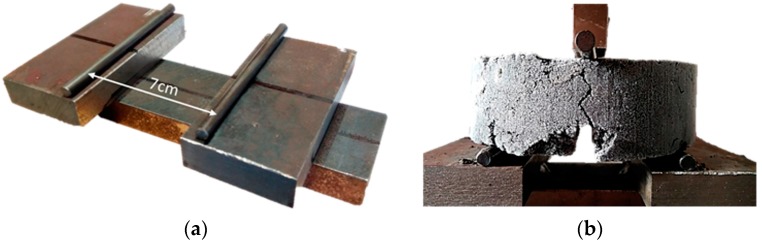
Details of the (**a**) Cradle with 7 cm between supports; (**b**) Three point bending test.

The test started by freezing the specimens. The fact that they were frozen made the break more fragile. Therefore, the specimen was broken into two pieces. In this particular case, the specimens were kept in the freezer for at least 24 h to ensure that the specimen core was also frozen. Once frozen, the specimens were broken through a three-point bending test in an ad-hoc manufactured cradle with 7 cm between supports (see Figure 5a). The specimens were placed for testing, which is illustrated in Figure 5b. This corresponds to the most suitable configuration, according to the type of support provided by the radial saw used. Although this setup differs from those used in previous studies [8,11], the comparative nature of the test guaranteed its validity since the specimens were subject to the same conditions before and after breaking and healing. The speed of the test was set at 10 mm/min and the fracture resistance of each sample was recorded with the load cell inserted in the compression machine ($Rt^{bh}$). After breaking the specimens, they were stored in a room at 20 °C for two hours.

**Figure 6:**
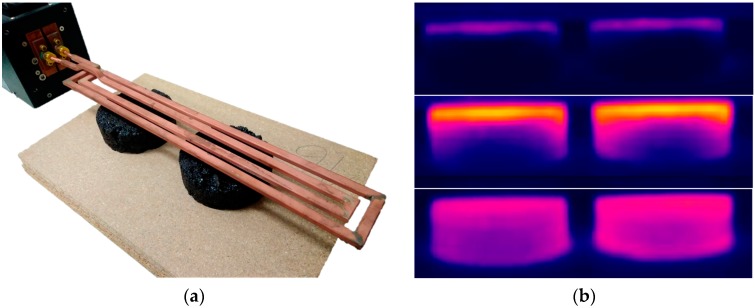
(**a**) Healing of specimens after magnetic induction; (**b**) Thermal images of the specimens.

After tempering, the specimens were subjected to magnetic induction at a distance of 1 cm from the coil and with a frequency of 329 KHz, an intensity of induction between 200 A and 600 A, and times between 90 s and 300 s (see Figure 6). The specimens rested 24 h after induction and then were frozen once more for 24 h. Lastly, the specimens were broken again with the same parameters used before, which enabled us to determine the fracture resistance after healing ($Rt^{ah}$) and the Healing Ratio (HR) (see Equation (2)). Considering that the geometry of the mixtures did not change before and after healing, HR could also be calculated by relating the load applied by the laboratory press before ($L^{bh}$) and after ($L^{ah}$) the process.

(2) HR=RtahRtbh= LahLbh

### 2.4. Statistical Analysis

A sequential combination of inferential and descriptive statistics was undertaken to support the interpretation of the results achieved by investigating and validating the conclusions drawn from them. The implications of the statistical tests considered, which are summarized in Table 2, were analyzed through the *p*-value of these results, according to a significance level of 0.05 [20]. 

Inferential statistics were applied to compare the performance of the different types of asphalt mixtures tested across the main parameters involved in the healing process. This branch of statistics was approached using either parametric or nonparametric tests depending on whether the values of the samples for the parameters implicated were normal and homoscedastic or not. The normality and homoscedasticity of the samples were checked through the Shapiro-Wilk [21] and Levene’s [22] tests, respectively. 

The use of descriptive statistics enabled the exploration of the relationships between the variables under analysis based on the results yielded by the inferential statistical tests, which could produce the grouping of some mixtures depending on their similarity. Since these variables were continuous, the Pearson correlation coefficient was applied to measure the strength and statistical significance of the linear relationships between parameters. 

## 3. Results and Discussion

This section presents and discusses the results achieved after conducting the experimental steps included in the methodology outlined in Figure 1. Therefore, determining the particle size distribution and temperature achieved by the by-products is listed in Figure 2. Those by-products prove to be suitable in terms of induction heating and were considered for the preparation of the asphalt mixture specimens in order to be healed in subsequent steps. The nomenclature used in this section for the by-products is consistent with that presented in Figure 2 such that asphalt mixtures were named by adding “_M” to the abbreviation of the by-product(s) compounding them, e.g., a mixture including SB3 and SB5 was SB3_SB5_M. 

### 3.1. Laboratory Tests

Table 3 gathers the particle size distribution (UNE 933-1) of all the by-products represented in Figure 2 except MB5 and MB6 whose curves were not calculated due to their shape. This could be dangerous for the grinders. These particular by-products were characterized only with the support of a measurement gauge, which yielded average diameters of 2 mm and 6 mm, respectively. 

One of the main premises of magnetic induction is that the smaller the ferromagnetic particle is, the more effective both their spread is and the achievement of homogenous temperatures across the mixtures. Nevertheless, the importance of the filler show that bitumen interaction limits the amount of filler that can be added to asphalt mixtures in order to not exceed the threshold that bitumen can accept. 

In addition to the particle size distribution, the other vital premise to facilitate healing by magnetic induction is the ease of the by-products to be heated, which depends on their ferromagnetic characteristics. The quantification of the thermal capacity of the materials enabled separating those by-products that were unsuitable to perform as heating inductors from those demonstrating to be valid for substituting virgin metal particles. The very first sift consisted of passing an ordinary magnet over the by-products in order to check their ferromagnetic potential aprioristically. This simple test allowed discarding some of them for their subsequent use as heating inductors such as S1, DS1, DS2, MS1, MS2, and MS3, which proved to lack enough magnetic attractiveness. Moreover, machining shavings MS5 and MS6 were not considered either due to their shape, which made them considerably difficult to mix with the other components and produced notable irregularities when the mixtures were compacted. 

**Figure 7:**
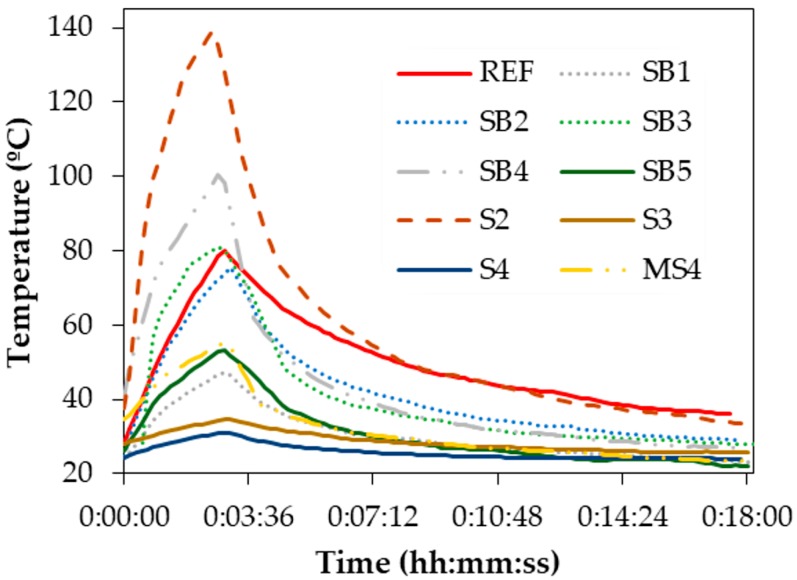
Temperature achieved by the by-products tested as potential heating inductors.

The remaining materials were tested, which was illustrated in Figure 3a. This yielded the results shown in Figure 7, which demonstrate that the magnetic induction barely affected some of the by-products such as S3 and S4. Their almost null influence by Eddy currents demonstrated they lacked enough metallic particles and/or they were too impure to be successfully heated. Similarly, SB1 and MS4 exhibited both a weak heat capacity and an unsuitable particle size distribution for manufacturing the asphalt mixtures. Although the bronze foundry slags reached the highest temperature, this peak was concentrated only in a few particles. Consequently, S2 was discarded because of its lack of homogeneity and the reduced number of metallic particles spread across their samples.

### 3.2. Mixture Dosage Design and Specimen Preparation

The design of the mixture dosage in the volume was preceded by the determination of the specific weight of the by-products selected in the previous step, which resulted in values of 7.850 g/cm^3^, 7.639 g/cm^3^, 7.465 g/cm^3^, 3.585 g/cm^3^ and 2.875 g/cm^3^ for REF, SB3, SB4, SB5 and S1, respectively. According to the nomenclature adopted for the by-products, the experimental mixtures were REF_M, SB3_M, SB3_SB5_M, SB4_SB5_M, and S1_SB3_M. Their bitumen contents and particle size distributions are shown in Table 4. 

Another important data point was the percentage of metal particles or by-products that compounded the mixtures. In REF_M, 5.0% of the mixture consisted of virgin steel grits (REF) while SB3_M, SB3_SB5, and S1_SB3_M contained 4.4%, 3.7% and 6.0% of sand blasting steel wastes (SB3). The latter two mixtures were also formed of 1.8% and 5.3% of sand blasting products (SB5) and green foundry slags (S1), respectively, which amounts to a total of 5.5% and 11.5% of by-products in the mixture. Lastly, SB4_SB5_M included 5.0% and 2.9% of sandblasting by-products (SB4 and SB5). Therefore, although the amount of inductors contained by each experimental mixture was different, their particle size distribution resembled that of REF_M, which enabled comparing the healing potential of virgin metallic particles and by-products in purpose-designed mixtures.

### 3.3. Healing Measurements Using a Break-Heal-Break Test

**Figure 8:**
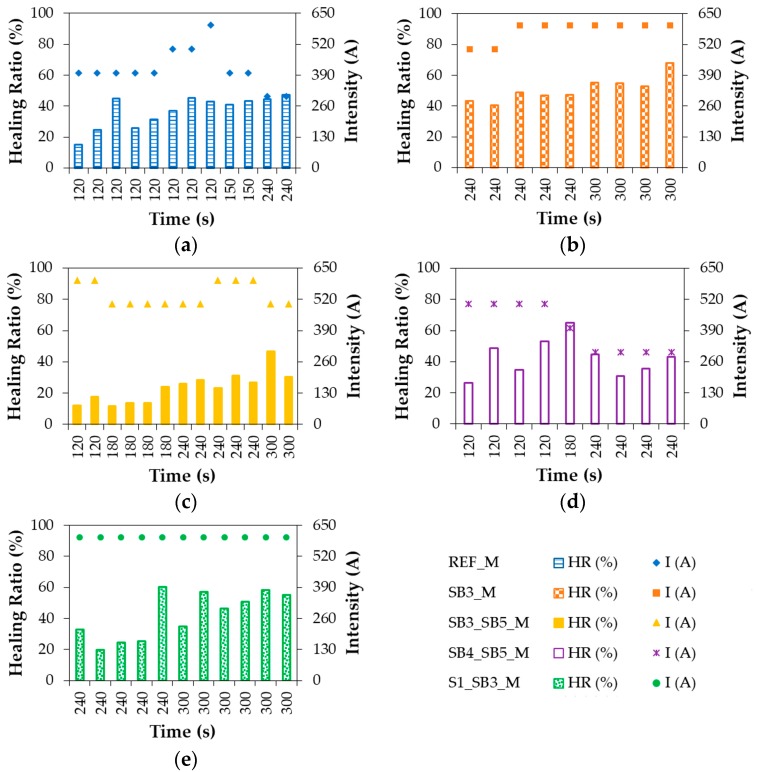
Healing ratios (%) achieved in relation to the time (s) and intensity (A) applied to test the different mixtures studied (**a**) REF_M; (**b**) SB3_M; (**c**) SB3_SB5_M; (**d**) SB4_SB5_M; (**e**) S1_SB3_M.

The break-heal-break test was applied to all the specimens of the five asphalt mixtures presented in Table 4, according to the methodology explained through Figure 5 and Figure 6 and Equation (2). The charts in Figure 8 illustrate the most relevant data collected from this test including the healing ratio (HR) achieved by the mixtures, the time (t) that the specimens were under induction (s), and the intensity (I) applied during this time (A). A time threshold of 300 s was established to prevent overextending the duration of the test in order not to compromise the efficiency of the healing process.

In the case of the REF_M (see Figure 8a), there were some specimens tested over 120 s with intensities ranging between 400 A and 600 A. The trend in these specimens was to achieve a better healing ratio for a greater intensity. However, the surface temperature achieved by the samples under 500 A or 600 A exceeded 150 °C, which is a value that could be dangerous for the bitumen properties. This fact led us to test other specimens using less intensity and more time such as those tested for 240 s and 300 A, which reached only 94 °C. However, this reached the highest HR for this mixture (47%). A notable increase in the healing ratio was also produced when keeping intensity at 400 A and changing time from 120 s to 150 s.

In contrast, the application of intensities of 300 A and 400 A did not heat enough the specimens of SB3_M, which led the values of intensity used with this mixture to increase. Therefore, significant values were obtained when applying 500 A during 240 s, which reached a healing ratio of about 42% (see Figure 8b). Surface temperature was around 90 °C, which enabled bitumen to flow (flow starts above 60 °C) without being burnt. Another group of specimens was tested by increasing intensity to 600 A and yielding a value of HR of 47%. Lastly, a third group of specimens was subject to the experiment during 300 seconds with an intensity of 600 A, which produced the best healing ratios with average values of 55%. In this case, the results suggested a clear and direct relationship between time and intensity with HR. Overall, it is important to mention that despite of the time invested on the test, the temperature reached for these samples was always under 130 °C. 

Figure 8c indicated that the influence of time on the healing ratio for SB3_SB5_M was higher than in previous cases, according to the differences between the results achieved with 180 s/500 A, 240 s/500 A, and 240 s/600 A. The best values of HR in this mixture were reached for 300 s and 500 A. However, they were far from those obtained for the remaining mixtures. The answer to this poor performance was found in the initial dosages where this mixture contained the lowest percentage of inductor particles, which were not enough to be properly spread over the mixture and made the healing process possible. 

SB4_SB5_M is highlighted by the variability of results achieved by the specimens tested under the same conditions (see Figure 8d). The healing ratios of the samples healed for 120 s and 500 A and ranged from 26 to 53% while those that healed for 120 s and 500 A resulted in values of HR from 30 to 45%. Therefore, the identification of which parameter was more influential on the healing of this mixture was difficult. Instead, the properties of these specimens were found to hinder the correct heat distribution over the mixture and the flowing of bitumen along the cracks. According to the values compiled in Figure 8d, the most effective combo for this mixture was 180 s and 400 A. 

Finally, S1_SB3_M (see Figure 8d) was tested using times between 240 s and 300 s and an intensity of 600 A. These parameters were used due to its similarities with SB3_SB5_M in terms of magnetic capacity and since both mixtures included sand blasting steel wastes as their main inductor. The general trend identified in the performance of these specimens suggested that the more time spent under induction, the higher the healing ratio is. The best results of HR for this mixture were about 60%. 

**Figure 9:**
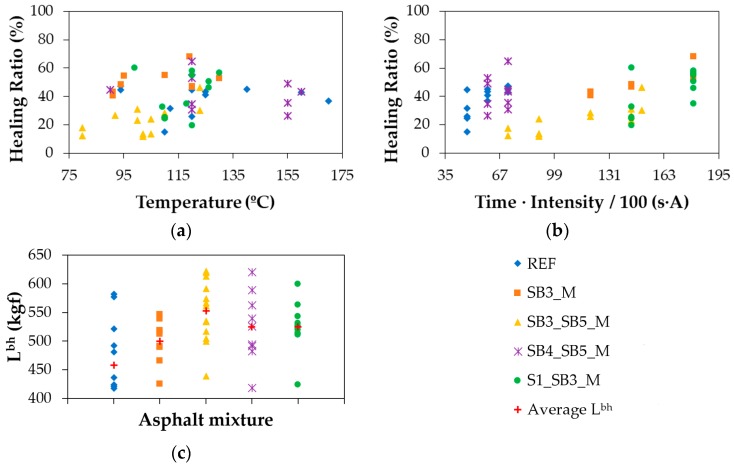
Healing ratios (%) achieved by the different mixtures in relation to (**a**) Temperature (°C) and (**b**) The product of time by intensity (s·A); (**c**) Breaking loads applied to the mixtures before healing ($L^{bh}$).

To further explore the variables affecting the healing ratios achieved in Figure 8 by the mixtures listed in Table 4, Figure 9 shows the relationship of HR with both the surface temperature achieved during the test (T) and the product of time and intensity (t·I). Both aspects were merged because the combined effect of exposure and magnitude was expected to be proportionally related to the healing ratios obtained, which is demonstrated in Figure 9a. On the contrary, the dispersion of the heating inductors across the mixtures was the reason behind the weak link between T and HR revealed in Figure 9b. Figure 9c shows the loads needed to break each sample before healing such that the average values rounded 460 kgf for the reference mixture and exceeded 500 kgf for the experimental mixtures with SB3_SB5_M providing the highest breaking resistance.

### 3.4. Statistical Analysis

The results represented in Figure 8 and Figure 9 were analysed in statistical terms by starting with checking the normality of the data associated with the parameters (t, I, t·I, T, HR) used to evaluate the five types of mixtures considered in Table 4. The application of the Shapiro-Wilk test demonstrated that only T was normally distributed for all the mixtures (*p*-value > 0.05 in all cases). However, its variance was heterogeneous according to the Levene’s test (*p*-value < 0.05). Therefore, the inferential analysis was carried out through nonparametric tests.

The Kruskal-Wallis test was used to evaluate all the mixtures at once, which resulted in the *p*-values listed in Table 5. This provided evidence of the statistically significant differences between the five groups (<0.05). In view of these results, the Mann-Whitney U test was applied to determine the absence or presence of significant differences for each pair of groups. The *p*-values compiled in Table 5 demonstrated that only two duos of mixtures (REF vs. SB4_SB5 and SB3 vs. S1_SB3) were statistically similar across all the variables involved (>0.05). On the one hand, the metallic purity of the steel shot wastes in SB4_SB5 along with the particle size distribution of this mixture explained its resemblance to REF. On the other hand, the affinity between SB3 and S1_SB3 was caused by the presence of steel grit wastes as main inductors in both mixtures since S1 lacked magnetic attractiveness. The remaining results indicated that the values of t, I, t·I, T, and HR reached by the other pairwise comparisons were significantly different in the majority of cases.

In light of the outputs of the inferential analysis, both REF and SB3_M were combined with SB4_SB5_M and S1_SB3_M due to their respective similarity such that the final number of groups to evaluate using descriptive statistics was three (REF + SB4_SB5_M, SB3_M + S1_SB3_M, and SB3_SB5_M). The quantification of the relationships between the variables involved in the healing process for these groups resulted in the Pearson correlation coefficients shown in Table 6.

These values suggested that the combined effect of time and intensity (t·I) provided the most solid positive correlation to HR. This showed that the longer and more intense the heating process is, the higher the healing ratio of the asphalt mixtures is. This coupled variable also explained the negative relationship between I and T for SB3_SB5_M, i.e., high intensities might not result in high temperatures unless they are applied for extended time periods. Therefore, heating time emerged as the most decisive parameter to ensure the healing of asphalt mixtures. The results in Table 6 also confirmed the uncertainty associated with the temperature achieved by the mixtures since this variable strongly depends on the distribution of the heating inductors across them, which hinders the existence of significant correlations for HR.

## 4. Conclusions

The outputs yielded by this investigation provided evidence of the potential for by-products to perform as heating inductors in asphalt mixtures. This was demonstrated through the application of a methodology consisting of the complete characterization in laboratory of a collection of diverse by-products supplied by metal industries, which enables their subsequent incorporation into asphalt mixtures that were tested in terms of healing capacity via magnetic induction. The set of by-products tested included slags, demolding sands, and materials from sandblasting and machining processes, which resulted in a varied sample of alternatives to compare with the virgin metallic particles contained in the reference mixture.

The results achieved in the different laboratory experiments revealed the limitations of some of the by-products used, which were unsuitable to be part of asphalt mixtures due to either their particle size distribution, their filler–bitumen interaction, or their heating capacity. According to these premises, the by-products selected due to their capacity as heating inductors were materials derived from sandblasting processes while green foundry slags were also added as a replacement of natural aggregates because of their lightness and particle size compatibility. The experimental mixtures required higher values of time and intensity than the reference one to reach satisfactory healing ratios except for the ones formed of steel shot wastes derived from sandblasting due to their similarity to the particle sizes of the virgin metals and the purity provided by this process. The relationships between the variables involved in the laboratory tests demonstrated that the greatest contributor for improving the healing ratios obtained was the time throughout which the samples were subject to magnetic induction. 

However, time is also the variable with the greatest influence when determining the feasibility of the healing of asphalt mixtures through magnetic induction since the process needs to be both fast and effective. Consequently, further work needs to be undertaken in the search for optimal combinations of time and intensity to boost the healing of mixtures containing different by-products. Another course of action to explore in the future relates to the improvement in replicating the characteristics of the cracks to be healed in real conditions since those tested in thelaboratory were larger and more concentrated. Nonetheless, the results presented in this research pave the way towards a more sustainable healing of asphalt mixtures by showing that the addition of by-products provides a viable solution for overcoming the economic and environmental drawbacks associated with the use of virgin metallic particles. 

## Figures and Tables

**Table 1:** Particle size distribution of the reference mixture.

**Sieve Size (mm)**	22	16	8.0	4.0	2.0	1.0	0.5	0.25	0.13	0.063
**Spindle Center**	100.0	95.0	67.5	42.5	31.0	23.5	16.0	11.0	8.0	5.0
**Top Limit**	100.0	100.0	75.0	50.0	38.0	39.5	21.0	15.0	11.0	7.0
**Bottom Limit**	100.0	90.0	60.0	35.0	24.0	17.5	11.0	7.0	5.0	3.0

**Table 2:** Summary of the inferential and descriptive statistical significance tests used.

Statistics	Type	Test
inferential	parametric	student’s *t* test (2 groups)
		one-way Analysis of Variance (ANOVA) (>2 groups)
	nonparametric	Mann–Whitney U test (2 groups)
		Kruskal-Wallis test (>2 groups)
descriptive	dependence	Pearson correlation coefficient

**Table 3:** Particle size distribution of the by-products considered.

By-Product	Sieve Size (mm)								
	16	8	4	2	1	0.5	0.25	0.13	0.063
REF	100.0	100.0	100.0	100.0	0.0	0.0	0.0	0.0	0.0
SB1	100.0	100.0	100.0	100.0	100	99.8	94.2	70.9	43.8
SB2	100.0	100.0	100.0	100.0	99.9	99.5	57.8	31.1	15.1
SB3	100.0	100.0	100.0	100.0	92.5	71.3	37.15	12.3	1.2
SB4	100.0	100.0	99.8	37.1	5.3	3.0	1.4	0.8	0.0
SB5	100.0	100.0	100.0	100.0	100.0	100.0	95.2	82.8	58.0
S1	100.0	100.0	98.2	71.7	28.6	6.7	1.8	0.7	0.0
S2	96.2	89.0	75.8	53.0	36.2	5.9	2.5	0.9	0.0
S3	100.0	100.0	100.0	0.0	0.0	0.0	0.0	0.0	0.0
S4	100.0	100.0	100.0	100.0	0.0	0.0	0.0	0.0	0.0
DS1	100.0	100.0	100.0	100.0	100.0	100.0	99.5	90.9	67.2
DS2	100.0	100.0	100.0	96.0	95.6	86.7	9.7	1.4	0.0
MB1	100.0	100.0	100.0	100.0	99.3	88.5	42.1	8.4	0.9
MB2	100.0	100.0	100.0	99.7	98.1	89.3	78.5	62.3	44.0
MB3	100.0	100.0	100.0	100.0	99.5	96.0	84.3	62.6	34.3
MB4	94.63	88.2	81.5	67.7	53.3	43.1	19.6	10.3	5.7

**Table 4:** Bitumen content and particle size distribution of the mixtures.

Mixture	Bitumen in Mixture (%)	Sieve Mize (mm)								
		16	8	4	2	1	0.5	0.25	0.13	0.063
REF_M	3.8	100.0	70.3	44.3	34.1	21.6	14.3	10.4	8.5	6.6
SB3_M	3.9	100.0	70.4	44.4	32.3	24.8	16.7	11.3	8.4	6.0
SB3_SB5_M	3.9	100.0	69.9	44.0	32.1	24.6	16.6	11.3	8.3	5.5
SB4_SB5_M	3.8	100.0	69.2	43.6	31.0	22.3	15.0	11.3	8.7	6.1
S1_SB3_M	3.8	100.0	70.9	44.6	32.3	24.9	16.8	10.6	8.4	6.9

**Table 5:** Comparative evaluation of the asphalt mixtures using nonparametric inferential tests.

Comparison	*p*-Value				
	t	I	t·I	T	HR
REF vs. SB3 vs. SB3_SB5 vs. SB4_SB5 vs. S1_SB3	0.000	0.000	0.000	0.011	0.000
REF vs. SB3	0.000	0.000	0.000	0.129	0.002
REF vs. SB3_SB5	0.007	0.001	0.000	0.011	0.007
REF vs. SB4_SB5	0.277	0.754	0.058	0.464	0.422
REF vs. S1_SB3	0.000	0.000	0.000	0.740	0.316
SB3 vs. SB3_SB5	0.030	0.126	0.014	0.647	0.000
SB3 vs. SB4_SB5	0.006	0.000	0.000	0.040	0.113
SB3 vs. S1_SB3	0.710	0.412	0.456	0.175	0.412
SB3_SB5 vs. SB4_SB5	0.292	0.003	0.000	0.007	0.001
SB3_SB5 vs. S1_SB3	0.011	0.009	0.001	0.011	0.004
SB4_SB5 vs. S1_SB3	0.002	0.000	0.000	0.131	1.000

**Table 6:** Pearson correlation coefficients between the variables involved in the healing process.

Interaction	Group		
	REF + SB4_SB5_M	SB3_M + S1_SB3_M	SB3_SB5_M
t∗T	−0.264	0.521 *	0.825 *
t∗HR	0.244	0.567 *	0.826 *
I∗T	0.504 *	0.585 *	−0.750 *
I∗HR	0.032	0.109	−0.105
(t·I)∗T	0.104	0.632 *	0.597 *
(t·I)∗HR	0.531 *	0.512 *	0.800 *
T∗HR	0.006	0.109	0.628 *

* Correlation is significant at the 0.05 level.
